# MESSAGE FRAMING AND INTENTION TO USE WEARABLE ROBOTS AMONG OLDER ADULTS: A PARALLEL AND MODERATED MEDIATION ANALYSIS

**DOI:** 10.1093/geroni/igae098.0113

**Published:** 2024-12-31

**Authors:** Clio Yuen Man Cheng, Vivian Weiqun Lou

**Affiliations:** The University of Hong Kong, Hong Kong, China (People’s Republic); The University of Hong Kong, Hong Kong, China (People’s Republic)

## Abstract

Little is known about the intention to use wearable robots among older adults, an emerging assistive technology to compensate for age-related functional decline. Informed by the prospect theory and senior technology acceptance model, we examined the effect of persuasive health messages in enhancing technology acceptance of wearable robots among older adults. A 2 (message framing: gain versus loss) x 2 (temporal framing: distal versus proximal) randomized experiment was conducted with 176 older adults aged 50 to 74 in Hong Kong. Non-parametric bootstrapping analyses were used to test the mediation model of attitudinal beliefs, performance, and perceived health benefits as parallel mediators of the relationship between message framing and behavioral intention to use wearable robots. Additionally, a moderated mediation model where the indirect effects of temporal framing are explored on perceived benefits in the mediation model. The results showed that gain-framed messages enhanced behavioral intention, through increased perceived health benefits (IE = -.242, 95% CI: LL = -.435 to UL = -.085, p <.01). Findings also provided evidence on the moderating role of temporal framing. Specifically, we observed that, for participants who were exposed to distally/gain-framed conditions, the IE is statistically significant (IE = -.301, 95% CI: LL = -.601 to UL = -.050, p =.032). For participants who were exposed to proximally/gain-framed conditions, the IE is higher and statistically significant (IE = -.432, 95% CI: LL = -.786 to UL = -.157, p =.007). This study suggests using proximally/gained-framed messages to increase wearable robots’ acceptance is promising.

